# From informed consent to adherence: factors influencing involvement in mass drug administration with ivermectin for malaria elimination in The Gambia

**DOI:** 10.1186/s12936-021-03732-z

**Published:** 2021-04-26

**Authors:** Alexandra Fehr, Claudia Nieto-Sanchez, Joan Muela, Fatou Jaiteh, Omar Ceesay, Ebrima Maneh, Dullo Baldeh, Jane Achan, Edgard Dabira, Bakary Conteh, Joske Bunders-Aelen, Tom Smekens, Henk Broekhuizen, Umberto D’Alessandro, Koen Peeters Grietens

**Affiliations:** 1 Athena Institute, Vrije Universiteit Amsterdam, Amsterdam, The Netherlands; 2 Unit of Socio-Ecological Health Research, Department of Public Health, Institute of Tropical Medicine, Antwerp, Belgium; 3 Medical Anthropology Research Center, Universitat Rovira I Virgill, Tarragona, Spain; 4 Medical Research Council Unit Gambia at London School of Hygiene and Tropical Medicine, Fajara, The Gambia; 5 Radboud University Medical Centre, Nijmegen, The Netherlands

**Keywords:** MDA, Adherence, Ivermectin, The Gambia, Malaria, Participation

## Abstract

**Background:**

The World Health Organization (WHO) recommends consideration of mass drug administration (MDA) for malaria control in low-endemic settings approaching elimination. However, MDA remains a controversial strategy, as multiple individual, social, and operational factors have shown to affect its acceptability at local levels. This is further complicated by inconsistent definitions of key indicators derived from individual and community involvement—coverage, adherence, and compliance—that cast doubts about the actual and potential epidemiological impact of MDA on disease control and elimination. This study aimed to identify limitations and enabling factors impacting involvement at different stages of a large cluster-randomized trial assessing the effect of combining dihydroartemisinin-piperaquine (DP) and ivermectin (IVM) in malaria transmission in The Gambia.

**Methods:**

This social science study used a mixed-methods approach. Qualitative data were collected in intervention and control villages through ethnographic methods, including in-depth interviews (IDIs), focus group discussions (FGDs), and participant observation conducted with trial participants and decliners, community leaders, and field staff. A cross-sectional survey was conducted in the intervention villages after the first year of MDA. Both strands of the study explored malaria knowledge and opinions, social dynamics influencing decision-making, as well as perceived risks, burdens, and benefits associated with this MDA.

**Results:**

157 IDIs and 11 FGDs were conducted, and 864 respondents were included in the survey. Barriers and enabling factors to involvement were differentially influential at the various stages of the MDA. Issues of social influence, concerns regarding secondary effects of the medication, costs associated with malaria, and acceptability of the implementing organization, among other factors, differently affected the decision-making processes throughout the trial. Rather than a linear trajectory, involvement in this MDA trial was subjected to multiple revaluations from enrolment and consent to medicine intake and adherence to treatment.

**Conclusions:**

This study went beyond the individual factors often associated with coverage and adherence, and found that nuanced social dynamics greatly influence the decision-making process at all phases of the trial. These issues need to be consider for MDA implementation strategies and inform discussions about more accurate ways of reporting on critical effectiveness indicators.

**Supplementary Information:**

The online version contains supplementary material available at 10.1186/s12936-021-03732-z.

## Background

Mass drug administration (MDA) is an intervention that aims at reducing the human reservoir of malaria infection by administering a full antimalarial treatment to the whole population, regardless of the individual’s infection status. This approach has been successfully implemented to control several neglected tropical diseases [1]. The World Health Organization (WHO) recommends the consideration of MDA for malaria control in endemic island communities and in low-endemic non-island settings approaching elimination, such as The Gambia, where there is minimal risk of re-introduction of infection, good access to treatment, and implementation of vector control and surveillance [2]*.* However, MDA remains controversial. A Cochrane systematic review concluded that MDA substantially reduces the risk of malaria infection, but few studies have shown a sustained impact beyond 6 months post-MDA [3]; further, MDA may increase the risk of selecting drug resisistance parasites [4]. Studies have also shown that achieving and sustaining high coverage is likely more important than the treatment used [5]. This requires the involvement of the target populations for as long as necessary in order to achieve the expected epidemiological outcomes, especially as a consistent trend of reduced MDA uptake over time, particularly in multidose regimens, has been observed [6].

Multiple individual, social, and operational factors have shown to influence acceptability and adherence to MDA. These include scepticism towards allopathic medicine [7], reluctance to undergo screening procedures like blood sampling [8] and pregnancy tests [9], concerns on drug adverse reactions [10], and reluctance of taking treatment without any symptoms [11]. Lack of clarity regarding the specific drug regimens administered [12], mobility [9], and mistrust between those who distribute the medications and local populations [13] have also been identified as factors decreasing treatment coverage and compliance. Implementing organizations also play a significant role in facilitating or limiting local involvement in MDA. Operational and implementation issues, such as providing adequate information, designing field staff supervision responsibilities, and preparing health systems for the intervention, heavily rely on management decisions and existing health policies at the national and local levels [14].

Lack of clarity regarding key MDA performance indicators such as coverage, compliance, and adherence contributes to the scepticism on MDA effectiveness. A systematic review on MDA strategies observed methods to estimate coverage and compliance are often not reported [7] or, when reported, definitions vary. Coverage may be estimated by taking the whole population [15], residents in smaller units of analysis (household or compounds) [16], or only those eligible for treatment [17]. Trials also differ on the amount of doses necessary to determine adherence to treatment. This can be as little as receiving treatment at any point in the intervention [17], completing a component of the treatment [18], or taking the full medication regimen [19]. The inconsistencies in the definitions and use of these indicators have a direct impact on the quality of the data collected, and, ultimately, on the arguments used to justify MDA implementation in already constrained health systems [20–22].

It has been recently suggested that ivermectin (IVM), commonly used for the control of onchocerciasis and lymphatic filariasis, may also play a role in malaria control and elimination. Indeed, IVM is toxic to *Anopheles* mosquitoes when they take a blood meal from a host that has recently received the drug. This provides the opportunity of killing mosquitoes that have escaped conventional vector control interventions [23], e.g. those that bite outdoors [24, 25]. Combining an efficacious anti-malarial with IVM may have a synergistic effect as the former would reduce the population parasite biomass and provide post-treatment prophylaxis while the latter would reduce vector densities [26, 27]. Eventually, IVM would reduce the minimal coverage required by MDA as mosquitoes, by feeding on several individuals over a short period, may also take a toxic dose of IVM from one of them. Transmission models suggest that adding IVM to a MDA intervention may interrupt transmission where standard MDA would be insufficient [28].

Factors influencing the decision-making process at different stages of an MDA implementation process, from enrolment to adherence, were investigated in the framework of a large cluster randomized trial (the MASSIV trial) assessing the effect of MDA with dihydroartemisinin-piperaquine (DP) and IVM in The Gambia.

## Methods

### The MASSIV trial

The trial was implemented in the Upper River Region (URR) in eastern Gambia, where the incidence of malaria is 1.7/PYAT [29]. This is an area of marked seasonal malaria transmission, with low vector density and high vector survival (parous rate 81–91% in URR as compared to 27–71% in other regions)[29]. Despite high coverage of standard control interventions—namely long-lasting insecticidal nets (LLINs), indoor residual spraying (IRS), Seasonal Malaria Chemoprevention (SMC), and artemisinin-based combination therapy (ACT)—URR is the Gambian region with the highest burden of malaria [30].

Thirty two study villages with a population between 140 and 700 each were identified in the south bank of the URR and randomly assigned to either the intervention or the control arm. Most villages were inhabited by a single ethnic group, the most common of which was Fula followed by Mandinka. Villages are led by an Alkalo, or village chief, and are made up of compounds, an enclosed space containing one or several households belonging to the same extended patrilineal family. The main economic activity is subsistence farming with a heavily reliance on remittances [31].

Prior to the MASSIV trial’s enrolment process, community sensitization meetings took place in all trial villages. Sensitization meetings were events where the trial and its details were introduced to the community by MRC field staff after receiving permission from the village Alkalo. The intervention was implemented with 3 MDA rounds per year, for 2 years, just before and at the beginning of the malaria transmission season. During each round, DP and IVM were administered as pills at the recommended dosage according to body weight over the course of 3 days. The primary endpoint was malaria prevalence determined by molecular methods at the peak of the second transmission season (November 2019) [32]. Data reported in this manuscript correspond to the first year of intervention.

### Study design

This social sciences study used a sequential exploratory mixed-methods design (QUAL ≥ quan) [33]. Initial qualitative research was implemented between July and November 2018, starting before the community sensitization meetings and lasting through the third MDA round. Data were collected in all villages, intervention and control alike, and informed the design of the quantitative component carried out in January–February 2019.

### Qualitative strand

#### Rationale

This strand focused on understanding the decision-making process for both those who accepted and declined being part of the trial, the trial effects on the residents’ daily activities, and the barriers and enabling factors facilitating uptake of the intervention at individual, family and community level.

#### Data collection

Initial qualitative research was conducted through ethnographic observations of all trial components, including sensitization meetings, the consent and trial enrolment processes, as well as treatment administration in all intervention villages. In-depth interviews (IDIs) and focus group discussions (FGDs) were conducted with the aid of trained field workers fluent in local languages.

#### Sampling strategy

Selection was purposive to allow for maximum variation and to further explore emergent themes. Community leaders, individuals who accepted or declined to take part in the trial, research team, and relevant stakeholders identified by the study team were included in this study.

#### Data analysis

Qualitative analysis was a continuous, flexible, and iterative process, where data were analysed in the field and further research was conducted to confirm or refute preliminary findings until saturation was reached. All discussions were recorded and transcribed verbatim by the research team. Raw data were processed in their textual form and coded to generate analytical themes for further analysis using NVivo Qualitative Data Analysis software (CITE software).

### Quantitative strand

#### Rationale

The survey focused on knowledge of the trial, decision-making processes around involvement, spread of trial-related information, and social influences, as well as perceived risks, burdens, and benefits. In addition, data were collected on the details and costs associated with the last time the respondents sought treatment for malaria. The questions and potential responses were framed upon the initial findings of the qualitative research strand.

#### Data collection

A cross-sectional survey was carried out in all 16 intervention villages and targeted individuals aged at least 12 years, regardless of level of involvement in the trial. Surveys were administered using Epi Info v. 7 by trained field workers using Android tablets. Data were synchronized with each tablet daily and regularly checked for quality. Once all surveys were conducted, data were exported into Excel to be analysed in STATA version 13.

#### Sampling

A sample size of 850 (rounded up to 900) was calculated in order to estimate an odds ratio of at least 1.5 as part of a multinomial logit regression for an outcome variable with 3 levels (no adherence, low adherence, and high adherence). However, during the analysis stage, the decision was made to combine no and low adherence into one category, resulting in binary logistic regression (no/low adherence and high adherence), which, incidentally, has lower sample size requirements. Per-village sample size targets were based on the proportion of the village’s population size to the total population of all intervention villages. Respondents were randomly selected from census data collected by the research team in November 2018. If a selected individual was unavailable or declined to be surveyed, another individuals of the same gender and age bracket was recruited from the census data.

#### Data analysis

Statistical analysis included frequencies of relevant variables based on survey responses and bi- and multi-variate analysis of selected variables to test for associations and predictors of the main outcome variables. To mirror the general criteria used by the trial, the analysis considered four endpoints:

1. Consent and enrolment: the proportion of surveyed individuals who self-reported providing written informed consent to participate in the trial (some of whom signed with their thumbprint);
2. Coverage: the proportion of surveyd individuals included in this study who stated having taken the trial medication at least once at any point during the MDA;
3. Self-reported adherence*:* the proportion of individuals self-reporting the number of days having taken the medication, classified as: (a) no/low adherence (0–6 times) and (b) high adherence (7–9 times);
4. Clinical card adherence: the proportion of surveyed individuals with (a) no/low adherence (0–6 times) or (b) high adherence (7–9 times) according to the clinical cards provided by the trial.

### Ethics

All respondents and their guardians (when under 18 years old) were explained the purpose of the study by field staff and gave informed consent or assent before being included in the qualitative or quantitative strands. Considering that the act of signing documents is not common practice for local populations and that it could produce mistrust towards the study team, we favored oral over written consent. Both studies were approved by the Institutional Review Board of the Institute of Tropical Medicine, Antwerp, Belgium, by the Scientific Coordinating Committee and the Gambian Government/MRC Joint Ethics Committee in The Gambia.

## Results

### Respondents characteristics

#### Qualitative strand

There were 157 in-depth and key informant interviews and 11 Focus Group Discussions (FGDs) conducted across the 32 villages. Respondents included village inhabitants, many of them holding community positions such as Alkalos, Chair of the Village Development Committees (VDC), traditional birth attendants (TBAs), village health workers (VHWs), and youth leaders. In addition, village residents, regardless of whether or not they took part in the trial, and MRC field staff were interviewed. Most respondents were women, belonging to the Fula ethnic group, and engaged in farming as their primary activity.

### Quantitative strand

The survey included 864 respondents across the 16 intervention villages. There were no refusals. The majority of respondents were female (66%), belonged to the Fula ethnic group (73%), and listed farming as their primary activity (80%). The median age was 29 (IQR: 19–41); 34% (294/864) reported previous participation in MRCG-affiliated research or programmes (Table 1).

**Table 1:** Demographic information of surveyed respondents

N = 864	n (%)
Age	
Median (IQR)	29 (19–41)
Mean (SD)	32.5 (16.1)
Gender	
Male	295 (34)
Female	566 (66)
Ethnic group	
Fula	626 (73)
Mandinka	172 (20)
Serahule	56 (5)
Other	9 (1)
Marital status	
Never married	240 (28)
Married	580 (67)
Separated/divorced	9 (1)
Widowed	31 (4)
Primary activity	
None	65 (8)
Farming	694 (80)
Herding	65 (8)
Business/trade	131 (15)
Domestic work	263 (30)
Other	56 (6)
Education	
None	357 (41)
Standard	243 (28)
Quranic	261 (30)
Household status	
Compound head	69 (8)
Household head	24 (3)
Compound member	226 (26)
Wife	360 (42)
Child	182 (21)
Other	2 (0)
Previous MRC experience	
Yes	294 (34)
None	547 (64)
Does not know	10 (1)
Does not remember	3 (0)

### Involvement in the MDA trial

#### Consent and enrolment, coverage, and adherence

**Fig. 1:**
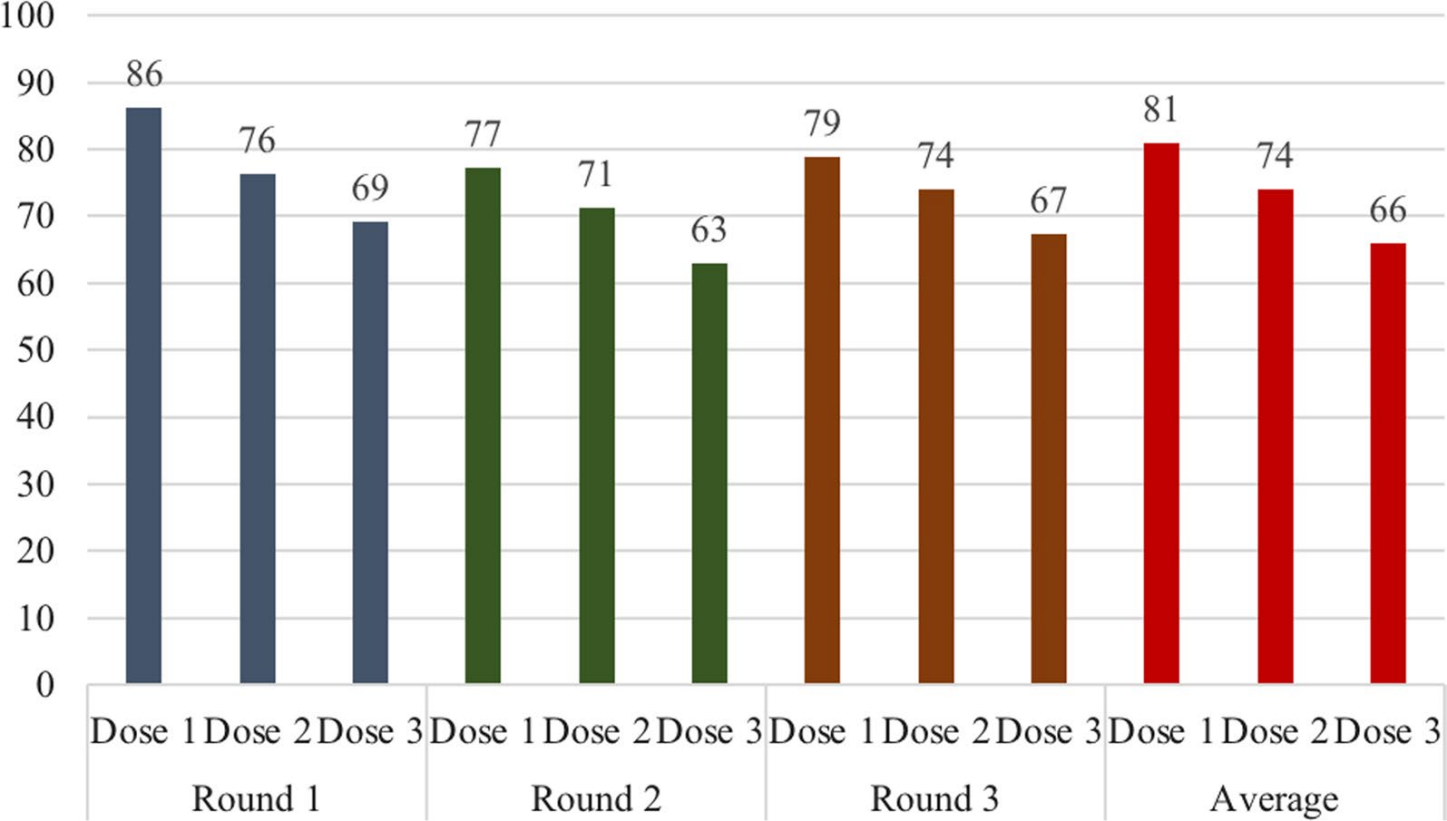
Percent of surveyed respondents with clinical cards who took trial medication by dose and round

Overall, 722 (84%) survey respondents self-reported to have provided written informed consent/assent and enrolled in the clinical trial; 70% (606) had taken the treatment at least once. Almost two third (62%, 534/864) of respondents self-reported no/low adherence and the rest (38%) high adherence. The trial clinical card was available at the time of the survey for 295 (34%) individuals; about half 45% (134/295) had evidence of low adherence (Table 2). It was possible, with the trial clinical card, to estimate adherence by round. The highest uptake was achieved by the first MDA round, followed by the third round. In addition, during each round, uptake decreased fron dose 1 to dose 3 (Fig. 1). Self-report of treatment (mean 6.9; SD 2.7) was very similar yet significantly higher than the information on the trial card (mean 6.5; SD 2.7) (paired ttest p-value = 0.001), though the values were highly correlated (0.7).

**Table 2:** Consent and enrolment, coverage, and adherence of surveyed respondents based on self report and clinical cards

	Of total surveyd n = 864 n (%)	Of those consented/enrolled n = 722 n (%)	Of those who took medicine 1 + times n = 606 n (%)	Of those with clinical card n = 295 n (%)
Based on self report				
Consent and enrolment	722 (84)	–	–	–
Coverage (1 or more doses)	606 (70)	606 (84)	–	–
No/low adherence (0–6 doses)	534 (62)	392 (54)	276 (46)	115 (40)
High adherence (7–9 doses)	330 (38)	330 (46)	330 (55)	173 (60)
Based on clinical card				
No/low adherence (0–6 doses)	134 (16)	133 (18)	130 (21)	134 (45)
High adherence (7–9 doses)	161 (19)	160 (22)	158 (26)	161 (55)

### Barriers to individual involvement in the MDA

Multiple factors affected respondents’ abilities or desires to take the trial medication. Some factors were more relevant at particular phases of the trial; others, such as farming responsibilities, were reported as relevant in all phases, from enrollment to adherence. Interestingly, of those who had enrolled in the trial, 16% (n = 142) did not take the medication. Some of them, though enthusiastic to enrol and promote uptake of the intervention among their communities, never intended to be treated:

“R: We went from one compound to another to inform them to come out [because] the MRC staffs have come.

I: Did you take the medication?

R: I did not take the medicine because of the reason I told you that after I take medicine it makes me sick and makes me vomit.” (Village TBA).

“R: Since they [MRC] start to give medication in the village I was the one working with them. I have not had anyone complain that he or she will not drink the medication anymore when the MRC people bring the medication.

I: Do you drink the medication?

R: No, I have not drunk the medication.

I: Why is it that?

R: You know since the MRC people came here, I am the one attached working with them. I had an emergency: one of my sisters was sick in [another village], her husband was not there, and I was the one responsible to take her to the health facility. Before I came back, I found that time has delayed, so that was the reason why I could not drink the medication.” (Male adult).

#### Travel/mobility

Having travelled or temporarily being away from the village was the primary reason for not enrolling in the trial (47% of those who did not enroll) or not taking all nine doses of the study medication (41% of those who took < 9 doses) (Table 3). Those in the age category 18–25 years were significantly less likely to take the medicine at all or have high adherence (Additional file 1: Table S1). When asked to elaborate on this finding, respondents explained that this age group was the least likely to take part on the trial because they travel for employment opportunities. Though some may return to assist in farming, young menwere the most likely to travel to the coast or abroad during the time of the MDA:

“No, I didn’t drink the medication. By then I was at the North Bank doing some electrical work. It seems like I am the guardian, but I don’t drink the medication because that very day didn’t find me here. But my wife and my children have all taken the medication.” (Village mobilizer).

**Table 3:** Reasons for not enroling in trial or completing full regimen based on surveyed respondents. Respondents could choose more than one reason

Reasons for not enrolling in trial	n = 140 n (%)	Reasons for not taking full regimen	n = 566 n (%)
Did not know reason for medicines	5 (4)	Did not know more than 1 dose/round	4 (1)
Pregnant	14 (10)	Did not know MRC was coming	10 (2)
Sick at time	6 (4)	Told to come later	4 (1)
Away from village	66 (47)	Ate before	1 (0)
Would not be here for MDA	1 (1)	Away from village	234 (41)
Busy at time	25 (18)	Too busy	96 (17)
Afraid of side effects	18 (13)	Side effects of medication	59 (10)
Healthy; does not need meds	2 (1)	Meds made others sick	12 (2)
Too much medicine	1 (1)	Too much medicine	11 (2)
Did not attend sensitization	2 (1)	Did not like taste	17 (3)
Does not like medicine	10 (7)	Got malaria	2 (0)
Medicines do not work	0 (0)	Took too much time	0 (0)
Did not want to fast	1 (1)	Other	34 (6)
Does not know	6 (4)	Does not know	7 (1)
No answer	1 (1)	No answer	5 (1)

Pleae see “Additional File 1: Table S1”

Being away from the village at the the time of the MDA round and time constraints related to economic activities—particularly farming—were also important reasons for not enrolling in the trial or not fully adhering to medication: 18% of those who did not enroll and 17% of those who did not complete it stated it was because they were too busy at that time (Table 3). This can be explained by the fact that the timing of the MDA and malaria season overlaps heavily with the rainy season, the most intense period of agricultural production in this region:

“Some people are working on their groundnuts and coos farms, this is what we depend on for survival. We have only two months remaining for the farming period to be completed so we need to work harder.” (VDC Chairman).

#### Implementation issues

Implementation issues impacting medicine uptake included misinterpreting or disagreeing with the conditions or logistics of the MDA, such as distribution times and locations, enrolment opportunities, and eligibility criteria. The requirement to fast for 3 h before and after medicine intake (for DP) was said to be an obstacle for their regular activities (such as farming) and, for some, was sufficient to refuse taking the medication.

#### Lack of privacy

As part of the eligibility requirements, women of reproductive age (15–49 years old) were required to undergo a pregnancy test. The implementation of the pregnancy tests varied by village, but it was often a source of concern due to privacy and other issues. In several villages, the location of the MDA lacked a private toilet facility and the results were often read at the same table where people registered. In some cases, women went home to collect their sample, but this required carrying an urine sample across the village. For many, particularly younger women and adolescents, this was a sufficient reason not to enrol or return for additional doses of the medication:

“We all [the entire compound] went to take the medicine, but I was asked to give my urine sample. I told them I am not married, and if it is about pregnancy, I know nothing has happened to me but they insisted that I must give my urine sample before they give me the medicine (…) Because the entire compound was there, and they asked me to give my urine sample, I refused because I am NOT having any relationship with any man, so I would not do it.” (Adolescent female).

“R: It [the pregnancy test] was done in the open [space], so people were sitting there. When you come and give them the urine sample, they will place it there and people will be sitting there looking at it (…) It should not happen like that.

I: And when did you decide to stop taking the medicine?

R: I stopped taking the medicine last month.

I: Can you tell us why? Anything that made you to stop taking the medicine?

R: Nothing happened, just on menstruation those days. That is why I did not go there.” (Adult female).

#### Knowledge of medicine and perceived side effects

Respondents’ personal beliefs also played a role in determining whether to enrol in the trial or continue taking the medicine. Some stated that they were too old for the treatment and others did not consider allopathic medicine as the most effective course of action for treating malaria:

“Well, it is a long time I don’t drink medication; I mostly depend on herbs.” (Village TBA).

Among those surveyed, 13% said they did not enrol and 10% said they did not fully adhere due to what they considered side effects of the medication (Table 3). Side effects reported by respondents during IDIs included excessive sweating, diarrhoea, vomiting, rash, dizziness, stomach pain, and burning chest or heart. Respondents reported having stopped the medicine intake as a result of personally experiencing or hearing of other community members with such symptoms:

“I have drunk the medication once, but I don’t continue drinking it anymore because since I drink the medication day before yesterday me and my wife vomited a lot, so this is why I will not go and drink the medicine today although I have finished drinking the medication in the first round.” (Adult male).

Specific concerns regarding the mosquitocidal effect of ivermectin were also mentioned:

You gave someone medication to take and when mosquito bites you that mosquito dies, it will also kill flies and lice and bedbugs… Will that not affect the person who took that medicine? That is the reason why some refuse.” (Alkalo).

However, though rarer, side effects were not always considered to be a negative consequence of the medication: some respondents described those symptoms as a clear sign that the medicine was having its intended effect.

### Enabling factors for individual involvement in the MDA

The most significant enabling factors at all stages included: recognizing malaria as a health concern, believing the trial’s benefits, and attending the sensitization meetings.

#### Recognizing malaria as a health concern

Both the qualitative and quantitative data showed that although there is a general perception that malaria has substantially declined in the last few years (63% of respondents), it is still considered the most serious health concern in the intervention area as stated by IDI respondents. Despite being a medically pluralistic population that uses Western biomedicine, herbalists and marabouts, respondents reported that going to health facilities is the preferred (87%) and most common option when they experience malaria symptoms for themselves (60%) or a child (44%) (Table 4).

**Table 4:** Trial beliefs and malaria health-seeking behaviors of survyed respondents

n = 864	n (%)
Believe malaria to be a problem	
Yes	220 (25)
Yes, but less now than in past	544 (63)
No	32 (4)
Does not know	35 (4)
No answer/missing	33 (4)
Non-health impacts of malaria, prompted, could choose multiple (n = 561)	
Costs of health facility	229 (41)
Costs of medicines	251 (45)
Costs of transport	215 (38)
Missed work	440 (78)
Missed school	359 (57)
Missed household responsibilities	374 (67)
None	6 (1)
Does not know	14 (3)
Other	2 (0)
Benefits to trial, unprompted, could choose multiple	
None	58 (7)
Access to study medicine	49 (6)
Access to medical personnel	7 (1)
Access to other medicines	4 (0)
Improved health	609 (70)
Access to transportation	1 (0)
Material benefits	1 (0)
Prevents malaria	389 (45)
Does not know	61 (7)
No answer	5 (1)
Benefit: access to medical personnel, prompted	
Yes	586 (68)
No	122 (14)
Does not know	81 (9)
No answer/missing	75 (9)
Benefit: access to transportation, prompted	
Yes	431 (50)
No	277 (32)
Does not know	84 (10)
No answer	72 (8)
Preferred treatment for malaria	
Nothing	7 (1)
Treat at home	27 (3)
Village health worker	7 (1)
Health facility	748 (87)
MRC	61 (7)
Other	3 (0)
Does not know	6 (1)
No answer	5 (1)
Treatment sought for last malaria: self, could choose multiple	
Nothing	1 (0)
Treat at home	23 (3)
Go to VHW	23 (3)
Go to health facility	518 (60)
Go to traditional healer	3 (0)
Go to MRC	26 (3)
Other	4 (0)
Does not know	7 (1)
No answer	1 (0)
Non-applicable	281 (33)
Treatment sought for last malaria: child, could choose multiple	
Nothing	0 (0)
Treat at home	10 (1)
Go to VHW	8 (1)
Go to health facility	383 (44)
Go to traditional healer	1 (0)
Go to MRC	12 (1)
Other	6 (1)
Does not know	1 (0)
No answer	0 (0)
Non-applicable	188 (22)

Inhability to perform income-generating activities was cited as the most common non-health impact of malaria (78%), followed by missing household responsibilities (67%), and missing school (57%). However, the costs associated with malaria medication (45%), visiting the health facility (41%), and transport to the health facility (38%) were also named as important consequences of malaria (Table 4). Furthermore, the total self-reported costs of the last malaria-related visit to the health center (the accumulating costs of medication, fees, food, and transport) were significantly associated with increased adherence to the trial for each outcome variable: those who paid more were more likely to enrol, take the medication at least once, and have high adherence (Additional file 1: Table S1).

Local communities examined the costs associated with malaria not only as the result of the direct investment necessary to treat it, but also as the cost derived from having to go to the health facility during the busiest time of the year:

“When [field nurse] and team come here, I gather all my family and ask them to go and take the medicine. I make sure all the children take the medicine for three times. I know I am comfortable when they are healthy. I will not be visiting the healthy facility always, I have been spending two, three hundred dalasi to buy medicine; therefore, when I am to stay healthy without paying a dalasi, do you think I will not take that seriously?” (Adult male).

“When the person develops any health problem is his own responsibility. From here to Basse the fare is seventy dalasi and whilst you are in Basse you cannot stay all day without food; therefore, if you people come all the way from Basse and bring us medication here if we accept taking the medication is for our own good and whoever refuses to take the medication refuses at his own detriment.” (Adult male).

#### Perceived benefits of the trial

The most common perceived benefits of participating in the trial were “improved health” (70%) and preventing malaria (45%). Believing on the trial’s benefits was significantly associated with higher levels of adherence in both bi- and multi-variate analysis (Additional file 1: Table S1). Though only a sparse number of respondents cited access to medical personnel or transportation as a benefit on their own, when prompted, 68% and 50%, respectively, said these factors were important trial’s benefits (Table 4).

“Our thoughts with MRC are that they are good and they have good medication, and if you are enrolled with the MRC, if you happen to get sick, they give you free transport from your home to the health facility. After treatment they send you home without paying anything and when they give you medication, you will not pay anything either.” (TBA female).

Receiving the medication was explained along the same line of thought, not necessarily as an individual health protective measure, but as a benefit some members of the family should receive on behalf of the other:

“My elder brother is a motor cycle mechanic. He works the whole day and before he comes back is already late. My father is also a farmer who goes and chases animals at the farms from morning until evening to make sure that the farm is not invaded by animals, and the other two are women that are in the compound are all breastfeeding, so they can’t come. That is why for me as I have time I came to participate in the MDA.” (Adult female).

#### Attending community sensitization meetings

42% of surveyed women and 38% of surveyed men (41%, n = 349, of all those surveyed) reported that they attended the sensitization meetings in their villages (Table 5). Based upon the data from those surveyed, 52% of the attendants were 26–49 years of age. Only 3% of sensitization meeting attendees were girls aged 12–17, many of whom reported during interviews that they were too busy with household responsibilities during the meeting times.

**Table 5:** Gender differences in social influence factors of surveyed respondents

Social influence	Men n (%)	Women n (%)	Total n (%)	p-value
Needed permision to participate	156 (60)	436 (82)	592 (75)	**< 0.0001**
Spouse took medication	121 (41)	195 (35)	316 (37)	0.066
Compound head took medication	158 (54)	334 (59)	492 (58)	0.11
Attended sensitization meeting	112 (38)	237 (42)	349 (41)	0.268

Although IDI respondents were not able to report many details about the information provided at the community sensitization meetings—and in some cases mentioned the meeting was held in a different language than the one spoken in the village—they were generally informed about the main focus (malaria) and activities (medicine distribution) of the trial, as well as the fact that they were not obliged to participate. IDI respondents also referred to the sensitization meetings as means to secure access to material benefits potentially available through their long-term collaboration with MRC:

*“*When studies like this are conducted in a village, try by all means to allow 3 or 4 people from your family to be part of the study (…) I said this because when there is future benefit for example this [other MRC trial in area], when your compound is not participating in that project you don’t benefit from this project.” (Adult male).

### Social influence

Social influence emerged as an essential factor in all phases of the trial—as both a barrier and facilitating factor. Respondents mentioned they waited to decide on whether or not to take the medication until after they saw the potential side effects it had on other people they know. Physical and social closeness within these villages facilitates this logic. Several respondents mentioned that when external visitors come to the village to introduce a specific intervention, they prefer to postpone their decision to take part on it until the announced services are actually provided, and they can learn from other villagers’ experiences. In this particular case, community members’ experiences with the first rounds of MDA influenced respondents’ decision-making processes subsequent rounds:

“Early on we were scared to drink the medicine because some people were not talking good about the medicine (…) Some were saying that the medicine is good, others were saying that if you drink the medicine it will make you vomit, dizzy, you can have diarrhea and weak body from it. This is what scared us in taking the medication at the initial stage (…) All this information comes out from our conversations because sometimes during our conversations you will meet with your good friends who will attest to you that this medication is very good, and it is very effective.” (Adult female).

Unanimously agreeing with a particular course of action was reported as a sign of cohesion at the village, compound, and household levels, in that order of authority and decision-making power. It was believed that these authority figures should act as caretakers and enforce their decisions as a way to benefit the entire village.

“Well in this situation if the whole village decides on something, I can’t dispute it. I will just agree to the decision made by the village leaders, because wherever the village stands that is where I will be.” (Adult male).

“When a whole village is doing something, and you are not doing the same, they consider you differently.” (Adult female).

IDI respondents often reported not having information about those not taking the medication, or referred to decliners as isolated cases or negative examples of community members that could be internally addressed:

“Yes, a few numbers of them didn’t want to participate in the study. (…) But in general, all the compounds are participating (…) the lost people whom you know that they are not educated, and they don’t listen to what the educated people are preaching to them, they are the people who spread bad rumours that MRC takes people’s blood (…) In any community those kinds of people exist. Even in our village, there are a few of them here, but people will not listen to them because everyone knows what is good and bad, and in such a situation we know how to handle such people in our community.” (VDC Chairman).

Although following the decisions of the majority is a well-established social norm, our data also show instances of disagreement among heads of household and village leaders. In those cases, heads of household used their authority to prevent members of their families from taking part in the study:

“I went to [the coast]. When I came back, my elder daughter, I saw the card with her, she went and drink the medicine. I asked my daughter, ‘How you got this paper?’ and she said, ‘it was giving to me by MRC people.” I asked the mother, the mother said she was not aware. I was very angry that time, I took the card from my daughter and I keep it. I told the mother that ‘I think I told you that I do not have interested of this program, so why in the absence of me you sent my daughter to go and take the medicine?’ So, I was angry.” (Adult male).

Having a spouse adhere to the trial was significantly associated with increased adherence for both men and women (Table 6). Women and minors, regardless of their age or marital status, were expected to consult with their husbands or caretakers (or their representatives in case of absence) about their potential adherence to the trial prior to providing informed consent. Women were significantly more likely to require permission to enrol in the trial and needing permission was significantly associated with increased adherence (Tables 5, 6).

“I: As a woman, do you agree with reason given by your household head about not to participate in the trial?

R: I will discuss with my husband, try to convince him to participate because it is good. When I discuss with him, if he agrees, we will join, but if he did not agree then I will not join the trial.” (Adult female).

“When they came for enrolment in this compound my mother was not around—she went to Basse to sell. The field worker found me here and asked me to come and enrol. I told him that I can’t give you my consent in the absence of my mother, because I need to seek consent from her first. Then the field worker reacted and said to me ‘you are a grownup person you can decide for yourself.’ I was not happy about that reaction from the field worker and I still insisted that I can’t give consent without my mother (…) Your elder is just your elder, and you know everyone has a position in a family, and for her, she is my parent. And the rest of the children, I am also their elder. Anything I am supposed to do I need permission from her. If she authorizes, I proceed with it; if she doesn’t give me permission, I stop it, so I can’t go beyond her decision.” (Adult female).

**Table 6:** Associations between social infleunce factors and outcome variables across genders. Odds ratio (95% confidence interval)

Social influence	Consent/enrollment		Coverage		Self reported: high adherence	
	Men	Women	Men	Women	Men	Women
Needed permission to participate	0.8 (0.4–1.6)	**4.4 (2.2–9.1)**	**1.3 (0.7–2.4)**	**3.7 (2.3–6.2)**	1.5 (0.8–2.6)	**2.12 (1.3–3.5)**
Spouse took medication	**3.5 (1.8–6.9)**	**2.6 (1.3–5.0)**	**2.5 (1.4–4.5)**	**1.5 (1.0–2.3)**	**2.6 (1.5–4.5)**	**2.3 (1.6–3.3)**
Compound head took medication	1.1 (0.6–1.9)	**2.0 (1.1–3.3)**	**1.9 (1.1–3.3)**	**1.5 (1.0–2.2)**	1.1 (0.7–2.0)	1.2 (0.8–1.8)

Bold values are statistically significant with a 95% confidence interval

Respondents mentioned their capacity to influence their social environment by advising others about the benefits of working with MRC, which was seen as a form of protecting the interests of the village. Acknowledging that elders act as role models for the village, fieldworkers strived to involve this sector of the population from early stages of the process by physically bringing them to their distribution point and having them motivate others to participate:

“Since the beginning of the MDA when the Alkalo and the village elders came, all the elders came as one group and the drugs were given to them (…) He was the first person who came (…) Because they see their elders taking it, why not them? I think this is the step they are following. Seeing their elders taking it, their grandfathers, fathers taking it in front of them, then they should be behind them.” (MRC Field Nurse).

### The role of MRC

MRC’s long-standing presence in the study area also held considerable influence in trial decision-making and adherence. Respondents expressed high appreciation for receiving—what they consider—health care in their own village and mentioned MRC’s presence as an important facilitating factor to increased access to health-care in general. This appreciation was reported to be retributed with unconditional support and trust in MRC activities. In fact, those who were first told of the trial by an MRC employee were significantly more likely to take the study medicine at least once (Additional file 1: Table S1):

“For me and my family, I will never step back when it comes to MRC work.” (VDC Chairman).

“Yes, I always tell my people to go and take the medicine, I tell them all the time, I always tell them that MRC does not give medicine that would make people sick; they give medicine to improve our health.” (Adult male).

In some cases, community members assumed a more active role and became advocates for the organization:

“My concern regarding the study is that there are people who know why they are drinking this medication, but there are others whom you know they are drinking this medication, but they don’t know why they are drinking this medication. They are just drinking it for the sake of drinking. So that being the case, I will take that responsibility before the second round. I will go and meet with these people within the compound and talk to them about why they are drinking this medication so that they will know. If not, in the second round when it is time for the MDA, they will start to take excuses by saying ‘I am going to the garden and other places.’ But if they are informed, the second round will be much better.” (Village mobilizer, male).

Negative past experiences, widely spread rumours regading MRC financially gaining from the sell of people’s blood, and questions regarding research practices also acted as arguments to decline involvement with this particular initiative:

“We were looking at MRC. Like when you participate in an MRC study, they will come for you, but when they find a sick person in the compound, they will not attend to the patient, but they will be interested in taking the healthy person and leave the sick one at home, and that is where we think is faulty. Secondly, we were thinking like when they recruit children in their study, they will take them and bleed them, and you know? People who eat sorrel don’t have a lot of blood. This was the problem. We started discussing with them all that, but they just passed us and left. We didn’t, this was the issue.” (Adult male).

## Discussion

MDA requires active individual and community involvement to secure efficacy of treatment [6, 7]. This mixed-methods study builds upon previous literature by showing that adherence to MDA is influenced by a multiplicity of individual, social, and implementation considerations constantly interacting and influencing decision-making at each and every stage of the MDA process. The results of this study show that rather than a linear trajectory, involvement is subjected to multiple revaluations from enrolment and consent to medicine intake and adherence to treatment [34, 35]. Issues of social influence, concerns regarding secondary effects of the medication, costs associated with malaria, and acceptability of the implementing organization, among other factors, differently affect decision-making processes throughout the trial.

The role that family and community members play on decision making is well known within The Gambian context [8]. These results support the notion that people’s level of involvement with and adherence to the trial is heavily influenced by the opinions, perceptions, and actions of their spouses, parents, compound heads, and community leaders. Respondents reported that unanimously supporting a particular course of action or decision made at the village level is a highly valued social norm; therefore, people are further influenced by social pressure to comply with what has been agreed upon by the community as a whole. Taking part in this particular intervention was described as an expression to support the communal decision of receiving the MRC and the trial medication—a decision that could render benefits not only for the individual, but also for the household, compound, and the entire village, both immediately and in the future [36].

However, this social influence is expressed differently between genders and across the different positions individually held within the village and the household. For women in particular, social factors were highly influential. Women who required permission to take part in the trial, for example, were significantly more likely to enrol, take the medication at least once, and self-report high adherence than those who reported not needing permission. Later on, however, women that initially accepted to enrol and attempted medicine intake, reported having stopped the medication due to a lack of privacy during the process of the pregnancy tests as it might elicit rumours within the village. Social influence acts in this case as a facilitating factor for enrolment, but also as a deterrent for further adherence to treatment. Similarly, having a spouse take the medication was significantly associated with increased enrolment, coverage, and high adherence for both men and women. However, men were still less likely to report high adherence, supporting previous arguments about men’s hesitations regarding MDA [37]. These findings highlight the importance of MDA community strategies that target gender-specific contextual factors to address the particular concerns and needs of the local population [6]. Beyond their direct impact on coverage and adherence, issues of privacy and autonomy should be analysed in their ethical dimension as predictors of inclusion among particularly vulnerable populations, such as women of child-bearing age, in implementation research [37–39].

Similarly, attending the sensitization meeting was found to be associated with consenting to enrol in the trial, taking the medication the medications at least once, and adherence to treatment. Although this association may be linked to the importance of information provision on individual decision-making, this conclusion is overly simple. For example, 60% of respondents sampled did not attend sensitization meetings, but 84% consented to enrolment in the trial. Attending the sensitization meeting loses significance with enrolment and coverage in the multi-variable analysis, demonstrating that other factors—such as perceived benefits—are concurrently involved in the decision-making process.

Previous studies have shown that the decision to take part in a trial in low-income settings often precedes information provision [40] and is made on the basis of non-health related arguments [41]. Expectations generated through the informal spread of information has been identified as particularly relevant, also in The Gambian context [8]. The political connotations of the sensitization process from the respondents’ point of view exemplify these dynamics. In this study, community members reported to use sensitization meetings to make themselves and their families visible in the village and MRC-supporting members, as this may render them eligible for any potential benefits. Additionally, by being present at the meetings, community members expressed their political power as part of an internal decision-making processes in the village and could demonstrate their position as supporters of the community’s decision.

Local village dynamics should also be considered while explaining the behaviour of those who enrolled in the intervention but had no intention of taking the medication, as well as those who were willing to just partially adhere. This can be interpreted as a non-confrontational and contextually-appropriate resistance strategy that allows villagers to remain autonomous in their personal decision-making in the context of power imbalances exising among community members and sparked by MRC’s presence. MRC’s particular influence and importance within The Gambia has been extensively documented [41–43] and this study supports this: respondents at large expressed trust in the organization and the desire to accept what they had to offer, particularly as they viewed essential services like transportation and access to increased medical care as benefits of the trial. This close interplay between individual decision-making and larger social dynamics should be considered when explaining enrolment and consent that does not translate into actual medicine intake [35].

Concerns about the potential side effects of the medication were reported as one of the main reasons to reject or interrupt treatment at different stages of the MDA. Although the data do not support a direct association between symptoms reported by the study respondents and the specific medications used in this MDA (all adverse events were reported regardless of their relation to the medication), it is important to point out that symptoms such as fever, headache, nausea, vomiting, dizziness, and itching have been previously reported for the medications used in this trial [15, 44]. Community concerns regarding safety of IVM’s mosquitocidal effect in humans should be seriously considered and adequately addressed in existing communication spaces with local communities, as they can overlap with those reported side effects and influence the uptake of and adherence to interventions.

Adding to the complexity of the decision-making process, the data support that the direct and indirect costs associated with having malaria (particularly the cost of the last malaria-related visit to the health facility) are of importance throughout all phases of the trial [11]. This continues to hold true when all other factors, such as social influences and individual demographics are considered. The possibility of receiving preventative treatment directly in their villages to avoid these expenses acts as an important justification to individually take part in the trial and motivate others, especially within the same family unit, to do the same.

There are important limitations in this study. At the moment of writing this manuscript, access to final figures of coverage and adherence for the general trial that could serve as reference to contrast the results has not been possible. Although this study only aimed to capture trends in coverage and adherence that could inform future MDA rounds, both coverage and adhrence data were below the ones originally targeted for this trial during the first year of implementation. The data hereby presented, however, cannot be extrapolated to the general trial as this study could not include elegibility criteria in the calculations.

There were also methodological challenges to accurately assess individual involvement in MDA. This study utilized two different approaches to assess the surveyed respondents’ consent and enrolment, coverage, and adherence: self-reported data and in-hand trial participation cards. Both approaches showed important limitations. In the case of self-reporting, it was difficult to frame survey questions in such a way that accurately responded to specific medication intake during each administration, in each one of the days that it was provided, and during the three rounds of MDA.The fact that this MDA included two different medications—often not distinguished by respondents—in each administration further complicated matters. The question was finally posed as “How many times did you take the medication during the MDA?”; this question cannot assess the sequence of intake or allow for us to calculate per-round coverage, but reflects the recollection of single times in which it happened. Field workers were trained on how to deal with questions or confusion from respondents. For the latter approach, difficulties derived from the possession of participation cards by village members. Many respondents reported that their cards were either lost or collected by MRC personnel after the trial. In addition, there were discrepancies between some respondents having self-reported not taking the medicine but having clinical cards with recorded doses. Although only minor differences emerged from the data collected through these different approaches, these discrepancies highlight the need to improve the data collection methods of trials and their concurrent studies to accurately measure progress towards elimination [21].

Of note, the word ‘participant’ was purposely avoided in this manuscript. The rationale for this decision is two-fold: first, there is a need to avoid any confusion between individuals involved in this sub-study (respondents) and individuals involved the trial in general; second, and more important, this study made evident that local populations have a broader understanding of what participating in a trial of this nature means. It can include activities such as being in touch with clinical teams to replicate information at the village level or cleaning and organizing the space where the medicine administration will take place. Since assessing participation in its political and social dimensions is beyond the scope of this paper, the decision was made to avoid the term and to report on this subject in upcoming manuscripts.

## Conclusion

Factors discussed in the previous sections demonstrate the complexity of interactions influencing adherence to multidose MDA regimens. From consent to medicine intake, local residents constantly revaluated their involvement with the trial based on multiple individual and social factors, including potential or actual side effects of the medication, the timing of the MDA in regard to economic demands, previous information regarding the intervention, as well as perceptions and experiences of other family members and community members in relation to the medication and its providers. In the same vein that authors have proposed to reimagine malaria treatment, diagnostics, and surveillance during this elimination era [45, 46], this study demonstrates the need to invest resources to improve critical indicators and more accurately report adherence to emerging elimination tools. In the case of MDA, it is essential that more complex ideas about individual and community involvement are incorporated in the understanding of the internal heterogeneity that could significantly limit the effectiveness of the intervention.

## Supplementary Information

**Additional file 1: Table S1. **Demographic, social, and implementation factors associated with consent and enrollment, coverage, and adherence in the MDA trial based on surveyed respondents.

## Data Availability

The dataset analysed for the current study is not publicly available due to confidentiality concerns; however, they are available from the corresponding author on reasonable request.
